# Degradation and Plant Transfer Rates of Seven Fluorotelomer Precursors to Perfluoroalkyl Acids and F-53B in a Soil-Plant System with Maize (*Zea mays L*.)

**DOI:** 10.1021/acs.jafc.1c06838

**Published:** 2022-07-15

**Authors:** Hildegard Just, Bernd Göckener, René Lämmer, Lars Wiedemann-Krantz, Thorsten Stahl, Jörn Breuer, Matthias Gassmann, Eva Weidemann, Mark Bücking, Janine Kowalczyk

**Affiliations:** † Department Safety in the Food Chain, German Federal Institute for Risk Assessment, Unit Feed, and Feed Additives, Max-Dohrn-Strasse 8-10, 10589 Berlin, Germany; ‡ Fraunhofer-Institute for Molecular Biology and Applied Ecology IME, Auf dem Aberg 1, 57392 Schmallenberg-Grafschaft, Germany; § Chemical and Veterinary Analytical Institute Münsterland-Emscher-Lippe (CVUA-MEL), Joseph-König-Strasse 40, 48147 Münster, Germany; ∥ Agricultural Technology Centre Augustenberg (LTZ), Neßlerstraße 25, 76227 Karlsruhe, Germany; ⊥ Department of Hydrology and Substance Balance, University of Kassel, Kurt-Wolters-Strasse 3, 34125 Kassel, Germany; # School of Chemistry, Monash University, Box 23, Victoria 3800, Australia

**Keywords:** PFAS, monoPAP, diPAP, FTOH, FTAC, transfer, fluorotelomer substances

## Abstract

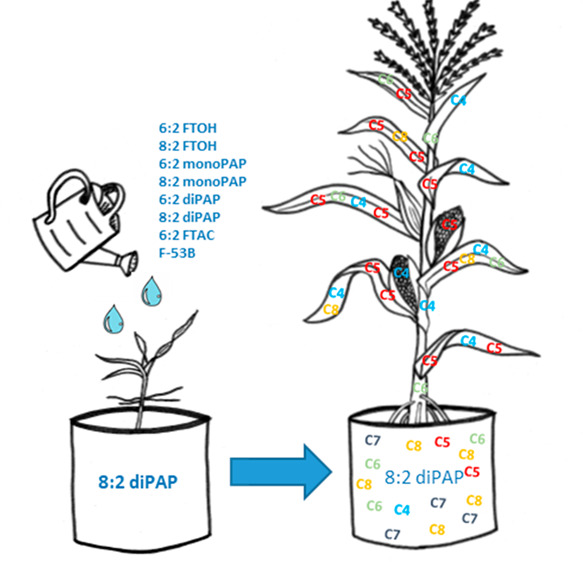

Fluorotelomer precursors in soil constitute a reservoir for perfluoroalkyl acids (PFAAs) in the environment. In the present study, precursor degradation and transfer rates of seven fluorotelomer precursors and F-53B (chlorinated polyfluoroalkyl ether sulfonates) were investigated in pot experiments with maize plants (*Zea mays L*.). The degradation of fluorotelomer precursors to perfluoroalkyl carboxylic acids (PFCAs) and their uptake spectra corresponded to those of fluorotelomer alcohol (FTOH) in terms of the number of perfluorinated carbon atoms. Short-chain PFCAs were translocated into the shoots (in descending order perfluoropentanoic, perfluorobutanoic, and perfluorohexanoic acid), whereas long-chain PFCAs mainly remained in the soil. In particular, fluorotelomer phosphate diesters (diPAPs) were retained in the soil and showed the highest degradation potential including evidence of α-oxidative processes. F-53B did not degrade to PFAAs and its constituents were mainly detected in the roots with minor uptake into the shoots. The results demonstrate the important role of precursors as an entry pathway for PFCAs into the food chain.

## Introduction

Per- and polyfluoroalkyl substances (PFAS), known as “forever chemicals”, are anthropogenic substances, which have been produced for over 50 years. Their chemical structure consists of a hydrophilic terminal group and at least one perfluoroalkyl moiety (C_*n*_F_2*n*+1_−), which is the most stable bond in nature and makes PFAS chemically and thermally inert.^1^ PFAS, which are used as surfactants, have a hydrophobic and a hydrophilic portion, which lowers the surface tension.^1^ Because of these unique properties, PFAS are used for over 200 categories of applications in almost all branches of industry and in many consumer products, for example, as water and stain repellents for textiles, food paper, and carpet impregnation, in fire-fighting foams, and for soil remediation.^2^ On account of the large variety of applications and their stability, PFAS are distributed in the environment worldwide.^3^ The OECD database of PFAS is listing more than 4700 PFAS compounds.^4^ The most prominent PFAS are the family of perfluoroalkyl acids (PFAAs) which includes perfluoroalkyl carboxyl acids (PFCAs) and perfluoroalkyl sulfonyl acids (PFSAs). PFAAs are divided into long-chain and short-chain. Buck et al. defined PFSAs from a chain length of six perfluoroalkyl carbon atoms (≥6 C) and PFCAs from a chain length of seven perfluorinated carbon atoms (≥7 C) as long-chain. PFCAs and PFSAs, which each contain a smaller number of carbon atoms, are classified as short-chain.^1^ PFAAs received the most attention because of their persistency and in some cases adverse effects on the health of humans and animals.^5^ Another substance class of PFAS are fluorotelomer substances. They are potential sources of PFCAs, which may have a significant impact as precursors on the PFCA concentrations detected in biological matrices.^1^ The degradation of fluorotelomer substances has been intensively discussed in the literature, wherein fluorotelomer alcohols (FTOHs) were reported as important intermediate metabolites.^6^ The subsequent degradation of FTOHs to PFCAs in different microbiological systems has been the object of *in vivo* experiments on trout^7^ and rats^8^ as well as in isolated hepatocytes of rats, mice, trout, and humans.^9,10^ The degradation pathway is comparable for all described models with the degradation of FTOH to PFCAs via a β-oxidation-like mechanism (oxidation of the β-carbon to form even-chain PFCAs).^7−12^ The α-oxidation (oxidation of the α-carbon to form odd-chain PFCAs) of 6:2 and 8:2 FTOH to perfluoroheptanoic acid (PFHpA) and perfluorononanoic acid (PFNA), respectively, has been observed in mammalian hepatocytes but is not expected to occur in the environment.^12^

The biodegradation of FTOH precursors has been described in different matrices, including soil. Soils are a sink for persistent pollutants like PFAS, as they may be exposed via natural (dry and wet deposition) and human (agricultural) activities.^13^ The following precursor substances were considered in this study because they likely emit into soil where they can enter the soil-plant paths and may degrade to PFAAs. FTOHs have been shown to oxidize to PFCAs of various chain lengths in house dust and have been considered as a likely atmospheric source of PFAAs which may also affect agricultural soils.^14^ FTOHs as degradation products are also formed by hydrolysis in sewage sludge and wastewater contaminated with fluorotelomer phosphates (PAPs).^11,15^ PAPs can occur as mono- (monoPAP), di- (diPAP), and trisubstituted polyfluoroalkyl phosphate esters (triPAP) with various fluoroalkyl chain lengths and are the primary products of polyfluoroalkyl surfactants used in (food) paper coatings and are thus one source of exposition to humans. This aspect, for example, has been confirmed in a study of two German colleagues (Halle and Münster) in which 4:2, 4:2/6:2, 6:2, and 8:2 diPAPs were detected in 10–46% of serum samples with concentrations in the range of <0.0002 to 0.687 ng/mL.^16^ Furthermore, PAPs were found in soil samples of the German Environmental Specimen Bank at concentrations of 1.75 ± 2.75 μg/kg soil dry matter (dm) and up to 1139 ± 700 μg/kg in samples of soil on incident sites.^17^ For the latter, a source of contamination has been PAP-containing paper sludge, which was applied as compost, for example, to field soils in Baden-Württemberg (Germany), led to the accumulation of PFAAs in the affected soils, to contamination of the cultivated crops, and to pollution of the groundwater.^18^ The PFAS-contaminated soils particularly in Baden-Württemberg contain diPAPs. These compounds are the primary products of polyfluorinated surfactants used in paper coatings and are hydrolyzed to one unit of FTOH as well as to one unit monoPAP during their degradation in aerobic soils.^11,15^ MonoPAPs are thus introduced either directly or as intermediate degradation products into the soil. Moreover, fluorotelomer acrylate polymers (FTACPs), used as dyes and dirt repellents for textiles, are high-molecular structures composed of fluorotelomer acrylate monomers (FTACs), hydrocarbon acrylate monomers, and other monomers.^19,20^ Fluorotelomer acrylates (FTACs) have been discussed as another PFCA precursor and have been detected in various matrices.^19^ The 8:2 FTAC has been shown to act as precursor of 8:2 FTOH and PFCAs in rainbow trout.^7^ Next to FTOHs, FTACs belong to volatile fluorotelomers which may be emitted into the air during manufacturing processes and enter the atmosphere from where they are transported over long distances.^21^ The 6:2 FTACs have been found at low concentrations in the air over the Atlantic and Northern Oceans and in rural air near Hamburg (Germany) (1.5 and 1.2 pg/m^3^, respectively).^21^ Therefore, the wet and dry deposition from the air into agricultural soils is a possible route for 6:2 FTAC into the soil-plant microcosm. Perfluoroctanesulfonic acid (PFOS) has been restricted under Annex I of the persistent organic pollution (POP) Regulation and Annex B (restriction) of the Stockholm Convention due to its concern on environmental and human health.^22,23^ The PFAS industry then shifted to replacement chemicals, such as F-53B as mist-suppressing agents in the chrome plating industry. F-53B is the trade name for 6:2 chlorinated polyfluoroalkyl ether sulfonate (6:2 Cl-PFESA) with 8:2 Cl-PFESA present as a minor component.^24^ It is mainly manufactured in China and was found in wastewater and surface water near an electroplating industrial park in Southeast China as well as in blood from Chinese people.^25^ Interestingly, F-53B was detected in herring gull eggs and bream liver samples in Germany, which indicates the worldwide distribution of PFAS.^17^

Many studies have investigated the pathways of PFASs, especially PFAAs, from aerobic soils to leaching and uptake into agricultural plants, as this makes them an important source of human exposure via drinking water,^26^ plant-based foods (such as cereals)^27^ and food-producing animals.^28^ Recently, Lesmeister et al. presented a review on the uptake of PFASs in crops and included studies on precursor degradation.^29^ Existing studies on precursor degradation in soil and PFAS plant uptake have generally considered only a few precursors in one study. The studies are often difficult to compare with each other due to different PFAS concentrations, different matrices (soils with varying properties, sewage sludge, hydroponically grown plants), different plant species, and plant parts (whole plant with and without roots). Bioaccumulation factors (BAFs) were partially used to describe the partitioning of precursor degradation products in soil and plants. Therefore, they relate the concentrations of one PFAA at one time point in the plant to the concentration in the surrounding environment (for example, soil or water). They do not include the degradation of precursors to various PFAAs over a time period.

There is insufficient knowledge about the biodegradation of precursors such as PFESAs, PAPs, FTOHs, and FTACs and their bioaccumulation in aerobic soils in the presence of plants.^29^ Therefore, the authors of the present study conducted pot experiments with maize plants (*Zea mays L*.). The soil was spiked with 6:2 and 8:2 FTOH, 6:2 FTAC, 6:2 and 8:2 monoPAP, 6:2 and 8:2 diPAP, and F-53B at a dose of 1 mg/kg soil fresh matter (fm). This dose corresponds to the level of PAPs on contaminated incident sites and is known to transfer at detectable amounts into maize plants. Maize was chosen as the experimental crop because of its worldwide use both as feed for livestock and directly as food for human consumption. The aim of this study was to investigate the biodegradation pattern of eight PFAS precursors and to quantify their sequestration and degradation products (PFAAs) in the soil as well as the transfer into the maize plant over the growth period.

## Materials and Methods

### Experimental Design

Two experiments in two different planting years were performed for the examination of the degradation of different precursors in a soil-maize system (Table S1). In May 2018, a greenhouse experiment with maize plants in Mitscherlich pots (soil capacity 15 L) was performed in the experimental station of the Landesbetrieb Landwirtschaft (LLH) in Kassel-Harleshausen (Germany). Eighteen kilograms of a PFAS-free reference RefeSol 01-A soil was filled into each of five pots per treatment as well as control pots without treatment. RefeSol 01-A is a standardized loamy sand soil with natural microbes, which is recognized by the German Federal Environment Agency (Umweltbundesamt) for test procedures in accordance with the German Federal Soil Protection Act.^30^ The physicochemical properties of the soil in dry matter (dm) were characterized as follows: pH 5.6, soil organic matter (OM) 1.55%, clay 6.1%, silt 17.2%, and sand 73.1%. The water holding capacity of the soil was 293 g/kg. Four maize seeds (variety DEKALB DKC 3941, FAO class 260, medium late-ripening) were planted into each pot and raised until the four-leaf stage. This maize variety is suitable for use as silage maize or grain maize in Central Europe, depending on the time of harvest. Subsequently, the maize plants were thinned to two plants per pot before the soil was spiked with PFASs. The stock solutions of the target PFAS were prepared to achieve a concentration of 1 mg/kg fm of soil. The substances comprised F-53B and the fluorotelomers 6:2 and 8:2 FTOH, 6:2 FTAC, and 6:2 and 8:2 monoPAPs (Table S1). For these solutions, 90 mg of each substance were dissolved individually in a mixture of 10 mL of 99.8% ethanol and 10 mL of demineralized water. Demineralized water was added to the solution to make up a total volume of 1 L, and the solutions were shaken manually for 2 min. Each solution was divided into five aliquots of 200 mL and applied onto the soil of each of the five pots per treatment. Finally, 2 L of PFAS free tap water was added to each Mitscherlich pot. One day after spiking, each pot was fertilized with 1.5 g of combination fertilizer Nitrophoska (EuroChem Agro GmbH, Switzerland) (15% N, 5% P, 20% K, 2% Mg, 8% S). To ensure natural temperature and light conditions, the roof of the greenhouse was left open to the atmosphere and was only closed during precipitation. The complete facility was protected with a mesh screen to keep animals, for example, birds from eating the grain. During the growth period, the soil was regularly watered to ensure optimal growth conditions. Water draining out of the pots was captured and reused for watering of the same pot in order to ensure a closed PFAS balance. The plants were allowed to grow 84 days in accordance with the growth period of silage maize under arable conditions. At the end of the experiment, the plants were harvested at a dry matter content of 26 ± 2%. All plants were chopped, weighed, and pooled to one sample per treatment for the PFAS analysis. The weights of the plant compartments were calculated subsequently using data of mean compartment weights for the 6:2 and 8:2 diPAP treatments of the following year 2019 (48% of total plant weight for stems, 8.5% for leaves, and 43.8% for cobs). Soil sampling was performed 10 days after harvesting using a bucket auger in two areas of the plant pot. Both samples were pooled together, homogenized, dried, and analyzed.

In May 2019, a similar experiment was performed under equal conditions with 6:2 and 8:2 diPAP. The authors of the present study decided to add diPAPs to the experiment and to optimize the experimental conditions in terms of weighing the plant compartments and analyzing the samples per compartment in triplicate. The pots were filled with 14.5 kg fm of RefeSol 01-A soil each and were spiked with 145 mL diPAP solution to reach a concentration of 1 mg diPAP/kg fm soil as in the previous description. For the 8:2 diPAP solution, the dissolution process with ethanol was not successful. Therefore, the dissolution was performed with isopropanol. However, in the 1 L volumetric flask, skin formation occurred on the surface of the liquid, which was again dissolved by adding small amounts of isopropanol shortly before spiking. After a growth period of 118 days, soil samples and plant compartments (stems, leaves, and cobs) were harvested, weighed, dried, and analyzed for PFAS.

The roots were washed thoroughly to remove the remaining soil particles and were rinsed with ultrapure water. The root weights were solely obtained for the diPAP treatments, and therefore the PFAS concentrations of the roots were partially included in the data analysis.

### Standards and Reagents

For application, 6:2 and 8:2 FTOHs (99.7% and 98.2%, respectively) and 6:2 FTAC (99.9%) were purchased from Fluorchem (Hadfield, U.K.) and 6:2 and 8:2 diPAP were custom-synthesized by the University of Giessen, Germany (purities >98%). F-53B was purchased and imported from China as a technical product from dgm China (Beijing, China). The 6:2 and 8:2 monoPAP were synthesized from the corresponding FTOHs as follows. A solution of the corresponding n:2 FTOH (14 mmol) and P_2_O_5_ (18 mmol, fractionated added) in acetone was stirred under reflux for 10 h. The ammonium salt of the phosphoric acid ester was precipitated with an ammonia solution (25% w/w). The crude product was rinsed with acetone to obtain the white fine-product. All analytical PFAS standards and internal standards were obtained from Wellington Laboratories (Ontario, Canada). Ultrahigh quality (UHQ) water was purified with a Purelab Ultra system from ELGA (Wycombe, United Kingdom). Liquid chromatography mass spectrometry (LC-MS) grade methanol from Chemsolute (Th. Geyer, Renningen, Germany) was used. Tetrabutylammonium hydrogensulfate (≥99%), ethanol (absolute, >99.8%), isopropanol (>99.5%), and ammonium acetate for LC-MS were purchased from Sigma-Aldrich (St. Louis, U.S.A.). Ammonia solution, sodium carbonate, and sodium bicarbonate (analytical grade) were obtained from Merck (Darmstadt, Germany), and methyl *tert*-butyl ether (MTBE) was purchased from Honeywell (Seelze, Germany). Dry ice was obtained from Linde (Dublin, Ireland).

### Sample Preparation

For the analysis of PFCAs, PFSAs, perfluorosulfonamides (FOSAs), diPAPs and F-53B, samples were prepared using an ion-pair reagent method. For this, plant samples were homogenized with dry ice using a commercial blender (Thermomix TM1 (Vorwerk, Wuppertal, Germany)) and 1 g of the homogenized sample and 1 g of soil were weighed into a 15 mL of polypropylene (PP) centrifuge tube. The mixture was then spiked with 100 μL of an internal standard solution (100 μg/L in methanol each) and 1 mL of a 0.5 M tetrabutylammonium hydrogen sulfate solution, 2 mL of a buffer solution (0.25 M sodium carbonate and 0.25 M sodium hydrogen carbonate), and 5 mL of MTBE were added. The samples were shaken for 30 min on a circular rotation shaker and centrifuged at 4700 rpm for 10 min. The organic phase was transferred to another 15 mL PP tube with a glass pipet and was evaporated to dryness in a stream of nitrogen at 40 °C. The residue was resuspended in 0.5 mL of methanol and was treated in an ultrasonic bath for 5 min. After centrifugation at 4700 rpm for 5 min, the extracts were then transferred into PP autosampler vials for subsequent analysis via ultrahigh performance liquid chromatography coupled with a high-resolution mass spectrometer (UHPLC-HRMS). Procedural blanks without sample material were analyzed concurrently with all samples to determine any possible contamination. The calibration consisted of 10 concentrations (0.1, 0.3, 0.5, 0.7, 0.9, 2, 4, 6, 8, and 10 μg/L) which were prepared from analytical standards. In the case of PFAS concentrations in a sample solution exceeding the calibration maximum of 10 μg/L, the solution was diluted accordingly. All detected PFAS concentrations were corrected by taking the recovery rate of the corresponding 13C-labeled or deuterated standard into account.

The methods were validated by spiking noncontaminated control samples with all analytes at LOQ and 10-fold LOQ level. Five replicates of each fortification level were analyzed. The recoveries were within acceptable ranges of 70–120%, and the relative standard deviations were less than 20%.

Both the target method and the TOP assay were examined in an interlaboratory comparison for different matrices and found to be suitable for quantifying PFAS.

Analysis of monoPAPs by separate sample extraction with alkaline methanol was not possible due to low or nondetectable signals of internal standards. This was presumably caused by low extraction efficiencies and matrix effects and did not allow a reliable quantification.

The analysis of 6:2 FTOH, 8:2 FTOH, and 6:2 FTAC was performed by Fraunhofer IVV (Freising, Germany) with accredited methods for plant material and soil. In the case of plant material, 1 g of the homogenized sample was spiked with the corresponding internal standards and extracted with hexane in an ultrasonic bath. The extracts were then cleaned up with silica solid phase extraction (SPE) columns and analytes were eluted with acetone. The eluate was then concentrated in a stream of nitrogen at 40 °C to a volume of approximately 1 mL and analyzed via gas chromatography coupled with mass spectrometry operated in positive chemical ionization mode (GC-PCI-MS). For analysis of fluorotelomer compounds in soil, 5 g of the homogenized samples were spiked with the internal standards. Five milliliters of methanol were added and the samples were shaken for 90 min on a horizontal shaker and treated in an ultrasonic bath for another 15 min. The extracts were then filtered with a mixed cellulose ester (MCE, Berrytec, Harthausen, Germany) membrane before analysis via GC-PCI-MS.

### UHPLC-HRMS Analysis

The UHPLC-HRMS analysis of PFCAs, PFSAs, FOSAs, diPAPs, and F-53B was performed as described by Göckener et al.^31^ Briefly, sample extracts were analyzed with a C18 reverse phase column on an Ultra Performance LC system by Waters Corporation (Milford, MA, U.S.A.) coupled to a Q Exactive Plus HRMS system (Thermo Fisher, Waltham, U.S.A.) operated in electrospray negative mode. The composition of the technical F-53B product was determined to be 89.6% 6:2 Cl PFESA and 8.2% 8:2 Cl-PFESA. The analysis and quantification of monoPAPs was conducted as described by Göckener et al. but the mobile phases were modified with 2 mM ammonium acetate and 0.01% ammonia to increase the ionization efficiency.^31^ A list of all substances analyzed by UHPLC-HRMS including their acronyms, exact masses, and the corresponding internal standard used for quantification is shown in Table S2. All limits of quantification (LOQ) are listed in Table S3.

### GC-PCI-MS Analysis

The analysis of FTOHs and FTACs was performed on a Trace GC coupled to a TSQ Quantum GC (both Thermo Fisher) with a RTX200 column (30 m × 0.25 mm × 1.0 μm) from Restek (Bellefonte, U.S.A.). Ionization was conducted with methane as a reagent gas. The quantification of FTOHs was performed with the corresponding internal standard while M10:2 FTOH was used for the quantification of 6:2 FTAC.

### Calculation of Substance Behavior

BAFs or bioconcentration factors (BCFs) relate the PFAS concentration in the plant to the PFAS concentration in the surrounding soil or water. They are frequently used for quantifying the transfer of PFAAs into plants.^29^ In order to quantify and represent the degradation of precursors to PFAAs and their transfer into the plants over the growth period, eqs 1 and 2 were derived from the BAF/BCF equation for the present study. The conversion rate (CVR) represents the biodegradation of precursors to PFAAs in the soil-plant system over the growth period and is the ratio of the sum (in μmol) of the degradation products in soil and plant (*n*(∑PFAA)_soil_ + *n*(∑PFAA)_plant_) per pot at trial end to the applied amount of precursor (*n*(Prec)_appl._) using eq 1. In this data evaluation, CVRs were solely calculated for soil and shoot due to missing root weights. A stoichiometric factor (SF) of 2 is applied in the case of the diPAPs because one molecule of diPAP can transform into a maximum of two molecules of PFCAs.^11^ In the case of the other fluorotelomers, the SF was set as 1. For a more descriptive presentation of the results, the rates were determined in percentage. The recovery of the applied precursors (RR_Prec_) at trial end is calculated by the ratio of remaining precursors to the applied amount of precursors per pot using eq 2.
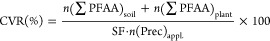
1
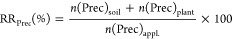
2The concentration rate in soil (CR_soil_) represents the amount of the remaining substances in the soil at trial end in relation to the applied amount of precursor and is the ratio of the amount of all PFCAs and precursors in the soil per pot at trial end to the applied amount of precursor per pot calculated using eq 3. The transfer rate into the plant (TR_plant_) of the applied substances and their degradation products was calculated on the same basis as CR_soil_ using eq 4. In the present study, the TR was calculated for the plant shoot only (TR_shoot_). Again, a stoichiometric factor of 2 was applied for the diPAP treatments. The total PFAS recovery rate for the entire soil-plant system (RR_total_) is the sum of formed degradation products and remaining precursors per pot or the sum of substances in the soil and shoots at trial end in relation to the applied amount of precursor per pot using eq 5.
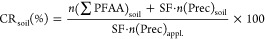
3
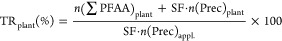
4
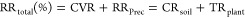
5

All equations are applicable for all individual substances and all matrices (for example, plant compartments).

## Results and Discussion

All plants grew without any noticeable deficits, that is, they did not show any abnormalities (for example, necrosis) or significant reductions in weight when compared to the control plants. The only exceptions were plants subjected to 6:2 diPAP, which had significantly lower weights (average 48.8 g (fm) less) than the control plants (*p* = 0.04) (Figure S1). At the end of the experiments, small concentrations of perfluorobutanoic acid (PFBA) in leaves (average 3.6 μg/kg fm) and of PFOA in soil (0.9 μg/kg fm) were detected in the control pots. The same levels were found in other experimental pots, where degradation to PFBA and PFOA would not be expected (for example, F-53B). This suggests a cross contamination with PFOA and PFBA, hence the concentrations of these substances in leaves and soil, and the measurement error quantity was subtracted from the PFAS contents in soil and leaves. Negative values were set to zero.

In the present study, all spiked fluorotelomers degraded to PFCAs which were found as persistent degradation products in all trial approaches. At trial end, a decline in the precursor concentrations was observed. The highest RR_total_ were observed for diPAPs and F-53B (Table 1). The 8:2 diPAP represents a large reservoir for PFCAs, since it presented both as the highest CVRs and RR_Prec_ among all of the fluorotelomers. For the other treatments, RR_total_ was found to be below 12%.

**Table 1:** Mean Conversion Rates to PFCAs (CVRs), Recovery Rates of Precursors (RR_Prec_), and Total PFAS Recoveries (RR_total_) at Trial End in Relation to the Spiked Amount of Precursor Substances in the Soil-Maize Plant System^a^

treatment	CVR (%)	RR_Prec_ (%)	RR_total_ (%)
6:2 FTOH	6.0	<LOQ^b^	6.0
8:2 FTOH	11.0	<LOQ^c^	11.0
6:2 monoPAP	6.6	n.a.	6.6
8:2 monoPAP	6.6	n.a.	6.6
6:2 diPAP	5.3	13.6	19.0
8:2 diPAP	23.8	43.2	67.0
6:2 FTAC	1.3	<LOQ^d^	1.3
F-53B	n.a.	134.4	134.4
6:2 Cl-PFESA	n.a.	82.4	82.4
8:2 Cl-PFESA	n.a.	52.6	52.6

a n.a. = not applicable

b LOQ was 5 μg/kg for 6:2 FTOH in soil and 20 μg/kg in plant material.

c LOQ was 5 μg/kg for 8:2 FTOH in soil and 25 μg/kg in plant material.

d LOQ was 10 μg/kg for 6:2 FTAC in soil and 21 μg/kg in plant material.

The low RR_total_ may be the result of the following: (1) undetected intermediates of the precursor degradation to PFCA (for example, fluorotelomer (unsaturated) carboxylic acids (FTCAs, FTUCAs)), (2) substance loss due to leaching or adsorption onto the pots and other equipment (for example, plastic bags for samples), (3) irreversible sorption onto soil particles and formation of nonextractable residues (NER),^32^ or (4) volatilization of precursors and intermediates. The study was conducted under natural conditions in order to simulate normal field growth (aeration, exposure to light). Because of this open system, volatile metabolites may have formed and could not be taken into account for the calculating of the RRs. Hence, this study focuses on the stable PFCAs, which were detected in soil and plant tissues.

### Overall Observations

**Figure 1:**
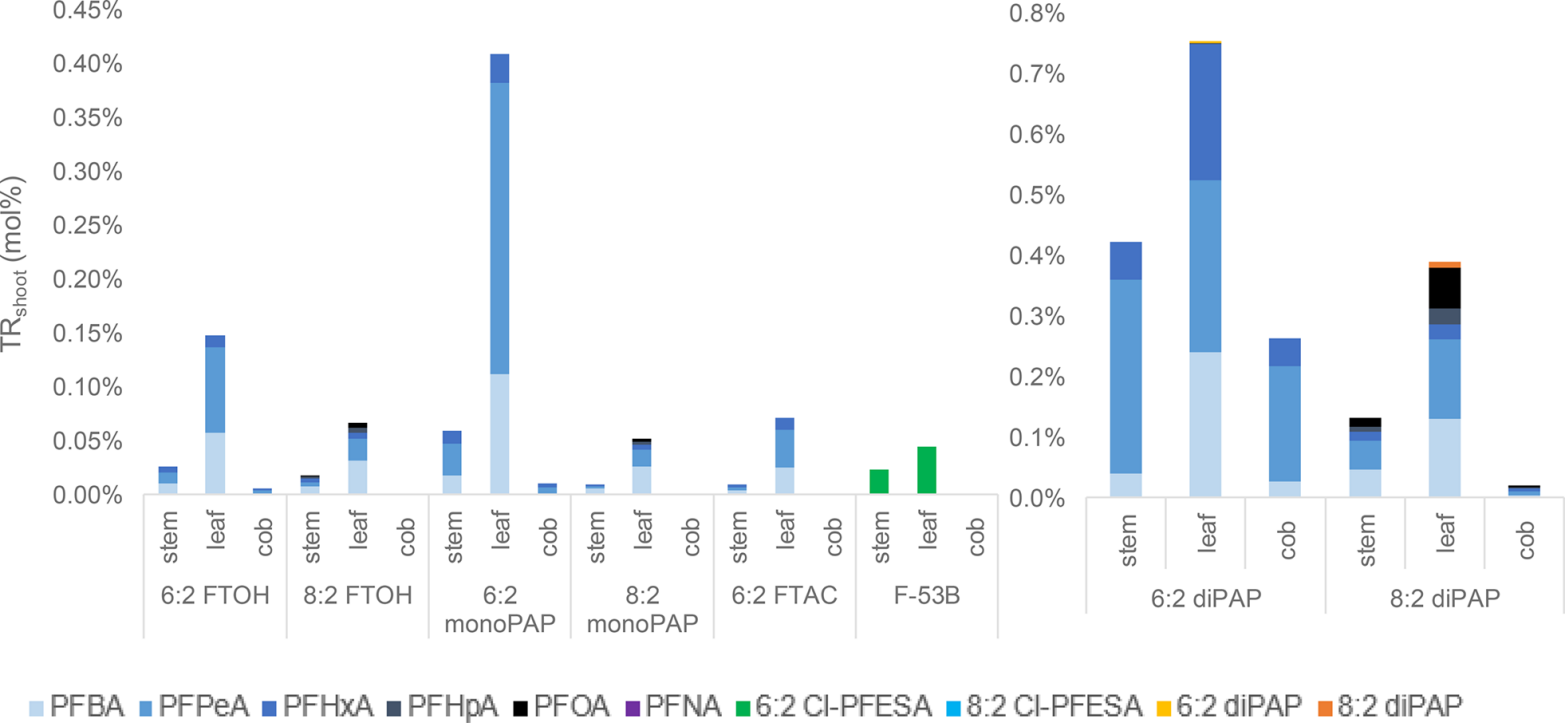
Concentration rates of PFASs in soil (CR_soil_) and transfer rates into the maize shoot (TR_shoot_) (mean values expressed in percent). Five plant pots with PFAS-free soil were spiked with 18 mg of precursor per pot before a growth period of 84 days. For 6:2 and 8:2 diPAP treatment, maize plants were exposed to 14.5 mg/pot before a growth period of 118 days.

At the end of the experiments, all spiked fluorotelomers were degraded to PFCAs, which accumulated in the soil and maize shoots (Figure 1). The majority of the degradation products, PFCAs of chain lengths C4 to C8, accumulated in the soil and their PFCA-patterns were similar between the treatments with fluorotelomers. Thereby, the fluorotelomers with six perfluorinated carbon atoms (6:2 fluorotelomers) degraded mainly to perfluorohexanoic acid (PFHxA) > perfluoropentanoic acid (PFPeA) > PFBA in the soil and showed higher TR_shoot_ values than the 8:2 fluorotelomers of the same substance class. Additionally, 6:2 diPAP was degraded to 0.01% PFHpA (0.7 μg/kg dm) in the soil and 0.001% PFHpA (4.3 μg/kg dm) in the leaves (Table S4). In the soils spiked with 8:2 fluorotelomers, the perfluoroalkyl chains of their degradation products were two carbon atoms longer than those of 6:2 fluorotelomers, that is, PFOA > PFHpA > PFHxA > PFPeA > PFBA. Liu and Liu arrived at the same conclusion in a study comparing 6:2 and 8:2 diPAP degradation in aerobic soils.^33^

**Figure 2:**
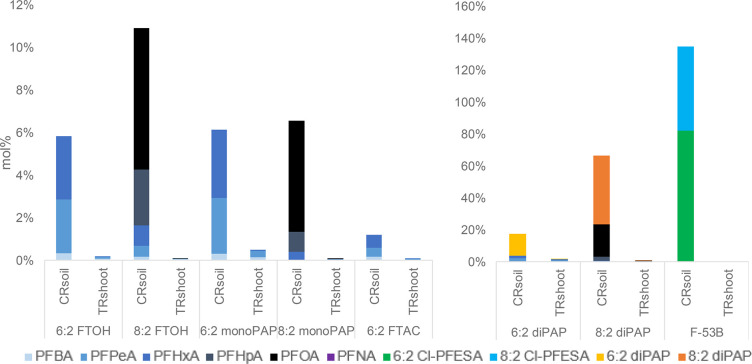
PFAS transfer rates (TR) into the shoot (plant above ground) and their distribution between the maize stems, leaves, and cobs (mean values expressed in percent). Five plant pots with PFAS-free soil were spiked with 18 mg of precursor per pot before a growth period of 84 days. For 6:2 and 8:2 diPAP treatment, maize plants were exposed to 14.5 mg/pot before a growth period of 118 days.

Treatment with 6:2 fluorotelomers resulted in higher TR_shoot_ values for short-chain PFCAs (≤C6) as a result of higher yields of short-chain PFCAs during the biodegradation process than with the corresponding 8:2 analogues (Figure 2). Short-chain PFCAs tend to transfer into the shoot due to their higher solubility and hydrophilicity.^34^ This is why the PFCA spectrum in the shoots of the present study shifts toward short-chain molecules in all treatments. Lower total yields of short-chain PFCAs in the degradation of 8:2 fluorotelomers lead to lower contents of short-chain PFCAs in the shoots compared to the 6:2 fluorotelomer treatments. Interestingly, in the shoots treated with the 6:2 fluorotelomers, PFPeA was found to be the main metabolite, whereas in the 8:2 fluorotelomer treated shoots, PFBA was mainly observed (Table S4). F-53B was not observed to degrade to PFAAs. The fluorotelomeric precursors themselves were not transferred into the shoots except for traces of 6:2 and 8:2 diPAP that were detected in the leaves.

The PFCA end products of all fluorotelomer treatments were mainly transferred into the leaves, followed by stem and cob (Figure 2). This is in line with the studies of different vegetables and grains grown on soil near a fluorochemical park in China.^27^ Additionally, differences in PFAS transfer into generative plant parts (grain) were investigated between wheat, triticale, and soybean plants with high PFAS transfer and rapeseed, barley and grain maize with comparably low PFAS transfer in the generative plant parts.^35^ In the present study, a quantifiable accumulation of short-chain PFCAs in the cob occurred in the treatments with 6:2 diPAP (0.3%) > 8:2 diPAP (0.02%) > 6:2 monoPAP (0.01%) > 6:2 FTOH (0.005%). The accumulation of largely PFPeA and PFHxA in the cobs were mainly found in 6:2 treatments, which might be caused by their higher degradation level to mobile short-chain PFCAs. Interestingly, PFBA was only found in the cobs of the diPAP treatments after a longer growth period. The developmental stage of the plant may affect the translocation of PFASs, especially PFBA, into the cob. To the best of our knowledge, there are no further data for uptake of PFAAs in maize, which might explain these observations. Further studies on PFAS accumulation in different plant growth stages will be necessary to confirm this possible explanation.

### Precursor Degradation Behavior

#### 6:2 and 8:2 FTOH

In the present study, 6:2 FTOH was degraded in descending order to PFHxA (3.0%) > PFPeA (2.6%) > PFBA (0.4%) in soil and shoot (CVR) (Table S4). In a hydroponic study with cultivated soybean plants, 6:2 FTOH was degraded to PFHxA (2.3%) and PFPeA (0.6%) after 144 h.^36^ PFBA was detected exclusively in the hydroponic solution of the soybean plants and was associated with degradation due to root-associated microbes. In the present study, PFBA was also detected in roots, stems, and leaves of the maize plants, which may be the result of the longer exposure period than in the soybean experiment. PFBA was also observed as a degradation product of 6:2 FTOH in a study on soil by Liu et al. (sandy loam, pH 5.8, OM 2.9%, 180 days).^37^ The authors suspected the formation of PFPeA (30.0%) and PFHxA (8.0%) as the main degradation pathway, whereas the formation of PFBA (2.0%) takes a secondary role. Furthermore, they assumed an alternative degradation pathway of 6:2 FTOH compared to 8:2 FTOH, because 6:2 FTOH was unexpectedly transformed mainly to PFPeA (loss of two perfluorinated carbon atoms) instead of PFHxA (loss of one perfluorinated carbon atom).^38^ This does not correspond to the observed degradation pattern of 8:2 FTOH to PFOA (loss of one perfluorinated carbon atom).^39^ The authors assume differences in steric hindrances of intermediate metabolites as 5:2 FTOH and 7:2 FTOH, which degrade further to PFHxA or PFPeA and PFOA or PFHpA, respectively. The results of this study show almost similar degradation rates of 6:2 FTOH to PFHxA (3.0%) and PFPeA (2.5%) in soil. Therefore, the authors of this study assume a balanced degradation of 5:2 FTOH to PFHxA (loss of one perfluorinated carbon atom) and PFPeA (loss of two perfluorinated carbon atoms). Differences in the degradation patterns are likely the result of different experimental conditions.

Analogous to results published in the literature, degradation of 8:2 FTOH could be observed in the present study. In soil and shoot, 8:2 FTOH was degraded to PFOA (6.7%) > PFHpA (2.6%) > PFHxA (1.0%) > PFPeA (0.5%) > PFBA (0.2%) (Table S4). In a hydroponic cultivation study with soybean plants, 8:2 FTOH was degraded to PFOA (6.0%), PFHpA (0.7%) and PFHxA (0.2%) after 144 h.^39^ The accumulation of metabolites in stems > leaves in the maize plant corresponds to the accumulation scheme in the soy plant. The amounts of PFHpA were 2.4 times higher in 8:2 FTOH spiked soybean root exudate controls than for PFOA, which was the main metabolite in the plant treatments. It was concluded that the degradation to PFHpA is primarily associated with the microbial metabolism stimulated by excretion of root exudates. The fact that PFHpA was not detected in unplanted soil by Wang et al.^38^ and the detection of PFHpA in soil, roots, stems, and leaves in the present study supports the theory of degradation of 8:2 FTOH to PFHpA by root exudate related microorganisms. In addition to PFHpA and PFHxA in the study with soybean plants, other short-chain PFCAs (PFBA, PFPeA) were formed in the maize plants described in this study. Equivalent degradation products (PFHxA and PFPeA) were observed in 10:2 FTOH degradation exclusively in the wheat roots of a pot study after 30 days.^40^ The authors suspect 10:2 FTOH and/or long-chain PFCAs are broken down via β-oxidation to short-chain PFCAs. The soil used in the experiment had a slightly alkaline pH of 7.7 (OM 4.1%), which might be the reason for the detection of PFHxA and PFPeA in the more acidic environment of the roots. In a study on the biodegradation of 8:2 FTOH in sandy loam (pH 5.8, OM 2.9%, 5 weeks), PFBA and PFPeA could not be observed in the absence of plants.^41^ Comparing the results of the experiments in this study (Table 2) with regard to planting and duration, root associated microbes in the acidic environment of the root exudates might influence the length of the FTOH degradation end products. The authors of the present study assume that the microorganisms present in the root exudates degrade soil-attached FTOH over time. An acidic soil pH seems to promote this process. More detailed studies on the influence of soil pH and root associated microorganisms on the degradation behavior of precursors will be necessary and will need to include further intermediate degradation products.

**Table 2:** Overview of the PFCA Metabolites of FTOH Degradation

precursor	study conditions	PFCA metabolites^a^	reference
6:2 FTOH	pot study on maize	C6, C5, C4	present study
	hydroponic soybean plants	C6, C5, C4	Zhang et al. (2020)^36^
	aerobic soil	C5, C6, C4	Liu et al. (2010)^37^
8:2 FTOH	pot study on maize	C8, C7, C6, C5, C4	present study
	hydroponic soybean plants	C8, C7, C6	Zhang et al. (2016)^39^
	aerobic soil	C8, C6	Wang et al. (2009)^38^

a In descending order

#### 6:2 and 8:2 MonoPAP

The degradation products and the distribution profiles in the plant compartments of the 6:2 and 8:2 monoPAP treatments were equivalent to the FTOH treatments and neither PFHpA nor PFNA were observed. To the best of our knowledge, there are no studies on the degradation of monoPAPs in systems with soil, wastewater treatment plant (WWTP) sludge, or plants. In a study by Lee et al., 6:2 diPAPs and monoPAPs were dosed in the aqueous phase of a mixture of raw wastewater and sewage sludge in closed bottles (anaerobic).^11^ The degradation of diPAPs to monoPAPs and subsequently to FTOH was described. The degradation end products of 6:2 monoPAP and 6:2 diPAP were PFHpA > PFHxA > PFPeA under anaerobic conditions. For the main formation of PFHpA, the authors assumed α-oxidation like processes as reported in studies on incubation of 6:2 FTCA^7^ and 8:2 FTOH^9^ in *in vitro* hepatocytes and of 8:2 FTOH in whole rats and isolated rat hepatocytes.^8^ The different experimental designs, including the anaerobic conditions, probably accounts for the different degradation end products compared to the present study. While the CVR in the soil of 6:2 monoPAP and 6:2 FTOH treatments are in the same range (PFHxA ≈ 3.5%, PFPeA ≈ 3.0%, and PFBA ≈ 0.4%), the PFCA accumulation in the maize shoot of the 6:2 monoPAP treatment is more than twice as high as in the 6:2 FTOH treatment (0.5% vs 0.2%). As other studies showed, FTOH was transferred into the plants where it would be expected to undergo further degradation to PFCAs.^39,40^ Therefore, it is conceivable, that the application of monoPAPs leads to higher FTOH levels and higher PFCA amounts within the plant than of the treatment with FTOH itself. In the 6:2 monoPAP treatment, the PFCA distribution in the plant compartments is in descending order: leaves (0.4%) > stem (0.1%) > cob (0.01%) (Table S4). In the 8:2 monoPAP treatment, PFBA (0.03%) and PFPeA (0.02%) were detected exclusively in the shoots,and not in the soil as in the 8:2 FTOH treatment. This suggests that the 8:2 monoPAP contingent may have been fully degraded and/or short-chain PFCAs in the soil may have been taken up completely by the plants or they were almost completely adsorbed onto soil particles as NERs. Small amounts of 6:2 diPAP in the soil of the 8:2 monoPAP treatment (2.5 μg/kg fm) were assumed to be an impurity of the applied substance standard and were not included in the data evaluation. Table 3 gives an overview on the degradation products of the monoPAPs of the present study and related literature.

**Table 3:** Overview of the PFCA Metabolites of monoPAP Degradation

precursor	study conditions	PFCA metabolites^a^	reference
6:2 monoPAP	pot study on maize	C6, C5, C4	present study
	dosed water (anaerobic)	C7, C6, C5	Lee et al. (2010)^11^
8:2 monoPAP	pot study on maize	C8, C7, C6, C5, C4	present study

a In descending order.

In this study, degradation amounts and pathways of monoPAPs and FTOHs of the same chain length are similar in the soil-plant system. We assume that existing differences in degradation rates and plant uptake levels can be attributed to different sorption and volatilization properties of the precursors. Further studies are required to elucidate degradation of monoPAPs and more sensitive analytical methods need to be developed for this purpose.

#### 6:2 and 8:2 diPAP

In the soil of the 6:2 diPAP treatment, PFHxA and PFPeA (1.7% each) were mainly detected with minor observations of PFBA (0.4%) and PFHpA (0.01%). These main degradation products were also observed in aerobic soils spiked with 6:2 diPAP^15,33^ and in a liquor mixture of wastewater and sewage sludge (Table 4).^11^ A minor formation of PFHpA was also found in this liquor mixture just as in the soil of pot experiments with *Medicago truncatula*.^15^ The authors of that study saw evidence of α-oxidation as reported in microbial and mammalian studies.^9,10^ In the present study, the degradation products of 8:2 diPAP were analogous to those of 6:2 diPAP with two carbons added. CVR for 8:2 diPAP in soil were in descending order: PFOA (20.2%) > PFHpA (2.3%) > PFHxA (0.4%) > PFPeA (0.2%) > PFBA (0.1%). This is in line with the observations of Liu and Liu, who investigated the biodegradation of 6:2 and 8:2 diPAP in soil under sterile and nonsterile conditions (unknown soil properties).^33^ They concluded that the biodegradation of 8:2 diPAP was slower compared to 6:2 diPAP due to lower degradation rates within 112 days of observation (Table 4). This is comparable to the decline to 13.6% for 6:2 and to 43.2% for 8:2 diPAP remaining in the soil of the present study after 118 days and would be equivalent to a first-order half-life of 33 days (6:2 diPAP) and 78 days (8:2 diPAP). The degradation products of 6:2 diPAP were transferred into the shoot at 1.4% (252.6 μg/kg dm) and reached the highest TR_shoot_ among all investigated precursors. In contrast, the degradation products of 8:2 diPAP were less than half of the amount accumulated in the plant shoot (0.5%, 78.0 μg/kg dm) (Table S4). The observed plant accumulation of PFPeA > PFHxA > PFBA > PFHpA is consistent with the metabolites, but inconsistent with the accumulation profiles previously reported for whole *Medicago truncatula* plants (including roots) by Lee et al. (Table 4).^15^ The inconsistent PFAA profiles in the plants might be caused by a higher spiking amount (100 mg 6:2 diPAP/pot), the different plant species, and the unknown soil properties in the study (5.5 month duration). Additionally, in the present study minor amounts of perfluorobutanesulfonic acid (PFBS) (1.9 μg/kg dm) were observed in the husks of the 6:2 diPAP treatments. This is assumed to be the result of cross-contamination with PFBS. In the present study, 6:2 and 8:2 diPAP were found in the roots (0.03% and 0.1%, respectively) and at minor concentrations of 10.8 μg/kg (dm) and 29.6 μg/kg (dm), respectively, in the leaves (0.003% and 0.009%), which is in total less than the uptake of 1% 6:2 diPAP into the *Medicago truncatula* plants of Lee et al.^15^ In a three month study with carrots, 8:2 diPAP was observed to be transferred into the leaves and peels but not into the core.^42^ Carrots are taproots and can therefore be compared to maize roots in terms of function as nutrients are transported acropetally via the transpiration stream within the plant (xylem). It can be assumed that diPAPs, on the one hand, adsorb onto the root and, on the other hand, are transported in small amounts via the root peel into the shoot and become stored in the leaves. However, further investigations on the diPAP transfer will be required to determine whether diPAPs are present in the plant due to soil-plant transfer or because of contamination. The metabolites from 8:2 diPAPs in the maize shoots of this study were similar to those in carrot leaves grown on compost amended soils (PFOA > PFHxA > PFHpA) (Table 4).^42^ In that study, low concentrations of PFNA, which is associated with the loss of one perfluorinated carbon atom, were detected (approximately 1 μg/kg in soil, carrot core, peel, and leaves). Further studies with 6:2 diPAP in sewage sludge^11^ and 8:2 fluorotelomers in microbial and mammalian systems^9,10^ observed further effects of α-oxidation processes. In the present study, degradation products associated with α-oxidation-like degradation processes were found after treatment with 6:2 and 8:2 diPAP. The degradation of 6:2 diPAP to 0.01% PFHpA in soil, roots, and leaves and of 8:2 diPAP to 0.0001% PFNA in the roots of one plant pot gives a further indication that these processes do not only occur in mammalian systems.

**Table 4:** Overview of the PFCA Metabolites of diPAP Degradation

precursor	study conditions	PFCA metabolites^a^	reference
6:2 diPAP	pot study on maize		present study
	soil	C6, C5, C4, C7	
	plant	C5, C6, C4, C7	
	pot study on *Medicago truncatula*		Lee et al. (2014)^15^
	soil	C6, C5, C4	
	plant	C4, C5	
	aerobic soil	C5, C6, C4	Liu and Liu (2016)^33^
	dosed water (anaerobic)	C7, C6, C5	Lee et al. (2010)^11^
8:2 diPAP	pot study on maize		present study
	soil	C8, C7, C6, C5, C4	
	plant	C8, C5, C4, C6, C7, C9	
	pot study on carrot		Bizkarguena et al. (2016)^42^
	soil	C8, C7, C6, C5, C4	
	plant	C4, C5, C6, C8, C7, C9	
	aerobic soil	C8, C7, C6	Liu and Liu (2016)^33^

a In descending order.

The diPAPs achieved highest CVRs to PFAAs considering maximum possible PFAA yields in the calculations (5.3% for 6:2 diPAP and 23.8% for 8:2 diPAP). High RR_Prec_ and comparably high PFCA-yields indicating a long-term availability of diPAPs, especially 8:2 diPAP, as a PFAA reservoir in the soil (Table 1). The contamination of field soils with diPAPs, as in Baden-Württemberg (Germany), therefore leads to a sustained contamination with a higher amount of different PFCAs in soil and plants compared to levels of monoPAPs and FTOHs.

#### 6:2 FTAC

In the present study, a conversion of 6:2 FTAC to PFHxA > PFPeA > PFBA (∑1.3%) was observed, which is very low compared to the CR of 6:2 FTOH (6%). Very low levels of PFCAs were transferred into the shoot (0.1%), mainly into the leaves, which is equivalent to the results of the 6:2 FTOH treatment. The 6:2 FTAC could not be detected at trial end neither in soil or plant. Royer et al. investigated the biodegradation of 8:2 FTAC in four different soil-types and observed half-lives (*t*_1/2_) of 3–5 days for 8:2 FTAC.^43^ Highest degradation rates to 8:2 FTOH (12.3%) were observed in the most acidic soil with the highest amount of organic matter (pH 5.3, OM 5.4%, 105 days). Subsequent degradation products (PFOA 10.3%, PFHpA about 3%, PFHxA about 0.4%) were equivalent to the 8:2 FTOH yield highest in the most acidic soil. This is again an indication of the importance of pH on degradation results in soil. In the present study, a much lower CVR was found in the soil of the 6:2 FTAC treatment compared to 8:2 FTAC, which probably implies a shorter half-life of 6:2 FTAC. The distribution profiles of PFCAs from 6:2 FTAC and 8:2 FTAC degradation are similar to those from 6:2 and 8:2 FTOH degradation, respectively (Table 5). The low CVR and the nondetection of 6:2 FTAC indicates the absence of a sufficiently large PFCA reservoir in the soil as seen in other precursors, and therefore no subsequent delivery of plant-permeable metabolites. It can be assumed that there is a substantial loss due to sorption (NER), volatilization, and/or degradation to undetected products in the soil. The degradation of 6:2 FTAC appears comparable to that of 6:2 FTOH as previous studies showed for 8:2 FTAC in trout,^7^ but more plant studies are required to evaluate the degradation behavior of this substance in soil plant-systems.

**Table 5:** Overview of the PFCA Metabolites of FTAC Degradation

precursor	study conditions	PFCA metabolites^a^	reference
6:2 FTAC	pot study on maize	C6, C5, C4	present study
8:2 FTAC	aerobic soil	C8, C7, C6	Royer et al. (2015)^43^

a In descending order.

#### F-53B

As previously reported in a study on PFOS-alternatives in different soils, F-53B was shown to be persistent and to not degrade to PFAAs.^44^ In the present study, F-53B did not degrade to PFAAs either. Its components 6:2 and 8:2 Cl-PFESA, however, were detected in the soil at high levels. Only the shorter-chained 6:2 Cl-PFESA was taken up into the shoot and was distributed within the stems and leaves (0.02% and 0.01%, respectively). Very high RR_total_ > 100% (134.8% for F-53B) were probably caused by the spot sampling of the soil. The 6:2 and 8:2 Cl-PFESA were found to be more readily adsorbed in soil with increasing OM-content, independent of pH and cation exchange capacity.^44^ Thereby, the rate of adsorption for 8:2 Cl-PFESA is higher due to the longer halogenated alkyl chain. In the present study, high CR_soil_ for 6:2 Cl-PFESA (84.4%) > 8:2 Cl-PFESA (52.6%) in the OM-poor soil are consistent with these findings. High root associated concentrations of 6:2 Cl-PFESA (2.6 mg/kg fm) and 8:2 Cl-PFESA (0.4 mg/kg fm) were detected compared to other precursors (next highest: 84.1 μg PFOA/kg fm in 8:2 FTOH treatment). The 6:2 Cl-PFESA was detected at low levels in the maize shoots (0.1%), whereas 8:2 Cl-PFESA was below the level of quantification (<LOQ) in the shoots (Table S4). Previously, 6:2 Cl-PFESA was found to largely accumulate in the roots with limited translocation into the shoots of hydroponically grown long-hair sedge (*Carex comosa)* on soil (OM 2.91%) during an 80 day growth period.^45^ These findings were also made in a 7 day hydroponic study on wheat seedlings, where a main accumulation of 6:2 and 8:2 Cl-PFESA in/on the roots and a minor uptake of 6:2 Cl-PFESA into the shoots were observed.^46^ In contrast to the present study, where no transfer of 8:2 Cl-PFESA into the maize plants could be detected, low concentrations of 8:2 Cl-PFESA were detected in the wheat seedlings. Because there are fewer apoplastic barriers (Casparian strip) and more transport proteins (aquaporins) in young parts of the root, it can be assumed that 7 day old wheat roots will result in higher uptake of molecules into the plant, such as 8:2 Cl-PFESA, compared to the full grown maize plants of the present study.^47^ As already investigated for diPAPs, the maize roots could represent a reservoir for PFAS as they are left overs in the field after harvesting.

In summary, the majority of the degradation products observed in the present study were consistent with previous investigations that have suggested fluorotelomer precursors degrade predominantly via a β-oxidation-like mechanism. Only for diPAPs, low concentrations of PFCAs related to an α-oxidation were detected as previously observed mainly in microbial and mammalian systems. In the present study, degradation of the investigated precursors to PFBA was observed to be irrespective of the number of their perfluorinated carbon atoms. In previous studies, the occurrence of PFBA was found almost exclusively in the degradation of the 6:2 fluorotelomers. PFAS precursor degradation was shown to be dependent on the plant species. In the experiments with applications of 8:2 diPAP to carrots and lettuce different degradation products were observed between the investigated species.^42^ While degradation products from PFBA up to PFNA (C4–C9) were determined in the carrot cultures in nutrient solution, only PFOA was detected in the lettuce cultures. Therefore, the study conditions have a strong effect on the degradation behavior of PFAS precursors (plant species, substrate type and properties, exposure duration, laboratory or outdoor conditions, application level) and thus strongly affect comparability between studies. These relationships regarding plant and soil properties will need to be addressed in future studies. It will be necessary to take into account the following uncertainties of the present study. Because of extensive root washing, which is associated with a loss of soil substance and fine roots,^48^ soil samples were taken by spot sampling in the pots and therefore the entire soil body could not be combined into one sample. Because of the different planting years and growth periods between diPAP treatments and the other precursor treatments, deviating TRs and CVRs may have been obtained. The used RefeSol 01-A soil is typical for soil under arable use in Central Europe. As the level of organic carbon in the soil can affect the transfer of PFAS from the soil to plants,^42^ our results cannot be easily transferred to other soils. Furthermore, future efforts should be made to improve the sensitivity of PFAS analytics and thus lower the LOQs especially for precursors, since precursors are mostly present at low concentrations in the soil (Table S3). Moreover, an extraction procedure for monoPAPs in different soil matrices should be developed to enable analysis of these molecules. Sum parameters, as the PFAS total oxidizable precursor (TOP) assay, can be involved to gain insight into the amounts of nondetected intermediate balancing. The observation of various contamination and organic matter levels of the soil as well as the influence on the plant uptake at different growth stages in conjunction with plant physiological processes will need further study. The role of root exudates should also be considered in relation to pH and different microorganism cultures.

Determination of PFAS concentrations at a single time point is unsuitable for quantifying precursor degradation (BAFs and translocation factors). It would be useful to level influencing factors (plant/soil and substance masses) by calculating degradation rates over time. In previous studies, precursor degradation rates were calculated on a molar basis, which facilitates the comparability due to exclusion of matrix weights on a dry or fresh matter basis. This study presents the equations for precursor degradation and thus provides a method to quantify the amount of degradation and pathways of PFAS precursors in a soil-plant system and contributes to the assessment of PFAS entry into feed and food.

## Supporting Information Available

Details on the experiments (Table S1), details on PFAS analyzed by liquid chromatography (Table S2), plant weights (Figure S1), limits of quantification (Table S3), and details on the data of the experiments (Table S4) (PDF)

## Abbreviations Used

BAF: bioaccumulation factor
BCF: bioconcentration factor
Cl-PFESA: chlorinated polyfluoroalkyl ether sulfonate
CR: concentration rate
CVR: conversion rate
diPAP: disubstituted polyfluoroalkyl phosphate esters
dm: dry matter
fm: fresh matter
FOSA: perfluorosulfonamid
FTAC: fluorotelomer acrylate
FTACP: fluorotelomer acrylate polymer
FTCA: fluorotelomer carboxylic acids
FTOH: fluorotelomer alcohol
FTS: fluorotelomer sulfonate
FTUCA: fluorotelomer unsaturated carboxylic acids
GC-PCl-MS: gas chromatography coupled with mass spectrometry operated in positive chemical ionization mode
LC-MS: liquid chromatography coupled with mass spectrometry
LLH: Landesbetrieb Landwirtschaft Hessen
LOQ: limit of quantification
MCE: mixed cellulose ester
monoPAP: monosubstituted polyfluoroalkyl phosphate esters
MTBE: methyl *tert*-butyl ether
NER: nonextractable residues
OM: organic matter
PAP: polyfluoroalkyl phosphate ester
PFAA: perfluoroalkyl acid
PFAS: poly- and perfluoroalkyl substance
PFBA: perfluorobutanoic acid
PFBS: perfluorobutane sulfonic acid
PFCA: perfluoroalkyl carboxylic acid
PFDA: perfluorodecanoic acid
PFHpA: perfluoroheptanoic acid
PFHxA: perfluorohexanoic acid
PFNA: perfluorononanoic acid
PFOA: perfluorooctanoic acid
PFOS: perfluorooctane sulfonic acid
PFPeA: perfluoropentanoic acid
PFSA: perfluoroalkyl sulfonic acid
POP: persistent organic pollution
PP: polypropylen
RR: recovery rate
SF: stoichiometric factor
SPE: solid phase extraction
TOP: total oxidizable precursor
TR: transfer rate
triPAP: trisubstituted polyfluoroalkyl phosphate esters
UHPLC-HRMS: ultrahigh performance liquid chromatography coupled with high-resolution mass spectrometry
UHQ: ultra high quality
WWTP: wastewater treatment plant
