# Supplementary material for: Degradation and Plant Transfer Rates of Seven Fluorotelomer Precursors to Perfluoroalkyl Acids and F-53B in a Soil-Plant System with Maize (Zea mays L.)
Source: J Agric Food Chem. 2022 Jul 15;70(29):8920–30. doi: 10.1021/acs.jafc.1c06838 (PMC9335875; doi:10.1021/acs.jafc.1c06838)
Supplement: Supplementary file 1 — jf1c06838_si_001.pdf [file jf1c06838_si_001.pdf]

Supporting Information

Degradation and plant transfer rates of seven fluorotelomer precursors to perfluoroalkyl acids and F-53B in a soil-plant system with maize (*Zea mays L.*)

Hildegard Just*†, Bernd Göckener‡, René Lämmer‡, Lars Wiedemann-Krantz‡, Thorsten Stahl§, Jörn Breuer||, Matthias Gassmann#, Eva Weidemann#, Mark Bücking‡ ††, Janine Kowalczyk†

† German Federal Institute for Risk Assessment, Unit Feed and Feed Additives, Department Safety in the Food Chain, Max-Dohrn-Str. 8-10, 10589 Berlin, Germany

‡ Fraunhofer-Institute for Molecular Biology and Applied Ecology IME, Auf dem Aberg 1, 57392 Germany

§ Chemical and Veterinary Analytical Institute Münsterland-Emscher-Lippe (CVUA-MEL), Joseph-König-Str. 40, 48147 Münster, Germany

|| Agricultural Technology Centre Augustenberg (LTZ), Neßlerstraße 25, 76227 Karlsruhe, Germany.

Department of Hydrology and Substance Balance, University of Kassel, Kurt-Wolters-Str. 3, 34125 Kassel, Germany

†† School of Chemistry, Monash University, Box 23, Victoria 3800, Australia

* Corresponding Author:

Hildegard Just (E-Mail: Hildegard.Just@bfr.bund.de, Phone: +4930 18412 28409)

Table S1: Overview of the experiments on precursor application on maize pots in two planting years

	First planting year 2018	Second planting year 2019
Applied substances	6:2 and 8:2 FTOH<br>6:2 and 8:2 monoPAP<br>6:2 FTAC<br>F-53B	6:2 and 8:2 diPAP
Growth period	84 days (silage maize)	118 days (grain maize)
Plant compartments	stem, leaf, cob	root, stem, leaf, cob
Sample preparation	single determination from homogenized composite sample	determination in triplicate per plant compartment
Maize variety	variety DEKALB® DKC 3941, FAO class 260	
Soil	RefeSol 01-A	
Dose	1 mg/kg soil (fm)	

Table S2: List of PFAS analyzed by liquid chromatography, their corresponding acronyms, exact masses of $[M\text{-}H^+]^-$ ions, and internal standards used for quantification.

Analyte (group)	Acronym Analyte	Chemical structure	m/z $[M\text{-}H^+]^-$	Acronym IS
Perfluorocarboxylic acids				
Perfluorobutanoic acid	PFBA	$C_4HF_7O_2$	212.9781	MPFBA
Perfluoropentanoic acid	PFPeA	$C_5HF_9O_2$	262.9749	MPFPeA
Perfluorohexanoic acid	PFHxA	$C_6HF_{11}O_2$	312.9717	MPFHxA
Perfluoroheptanoic acid	PFHpA	$C_7HF_{13}O_2$	362.9685	MPFHpA
Perfluorooctanoic acid	PFOA	$C_8HF_{15}O_2$	412.9653	MPFOA
Perfluorononanoic acid	PFNA	$C_9HF_{17}O_2$	462.9621	MPFNA
Perfluorodecanoic acid	PFDA	$C_{10}HF_{19}O_2$	512.9589	MPFDA
Perfluoroundecanoic acid	PFUnA	$C_{11}HF_{21}O_2$	562.9558	MPFUnA
Perfluorododecanoic acid	PFDoA	$C_{12}HF_{23}O_2$	612.9526	MPFDoA
Perfluorotridecanoic acid	PFTrDA	$C_{13}HF_{25}O_2$	662.9494	MPFDoA
Perfluorotetradecanoic acid	PFTeDA	$C_{14}HF_{27}O_2$	712.9462	MPFDoA
Perfluorohexadecanoic acid	PFHxDA	$C_{16}HF_{31}O_2$	812.9398	MPFDoA
Perfluorooctadecanoic aicd	PFODA	$C_{18}HF_{35}O_2$	912.9334	MPFDoA

Table S2 continued

Analyte (group)	Acronym Analyte	Chemical structure	m/z $[M-H^+]^-$	Acronym IS
<u>Perfluorosulfonic acids</u>				
Perfluorobutane sulfonic acid	PFBS	$C_4HF_9O_3S$	298.9427	MPFBS
Perfluorohexane sulfonic acid	PFHxS	$C_6HF_{13}O_3S$	398.9355	MPFHxS
Perfluoroheptane sulfonic acid	PFHpS	$C_7HF_{15}O_3S$	448.9323	MPFOS
Perfluorooctane sulfonic acid	PFOS	$C_8HF_{17}O_3S$	498.9291	MPFOS
Perfluorodecane sulfonic acid	PFDS	$C_{10}HF_{21}O_3S$	598.9227	MPFOS
<u>Fluorotelomer phosphate monoesters</u>				
6:2 Fluorotelomer phosphate monoester	6:2 monoPAP	$C_8H_6F_{13}O_4P$	442.9713	M2-6:2-PAP
8:2 Fluorotelomer phosphate monoester	8:2 monoPAP	$C_{10}H_6F_{17}O_4P$	542.9647	M2-8:2-PAP
<u>Fluorotelomer sulfonic acids</u>				
6:2 Fluorotelomer sulfonic acid	6:2 FTS	$C_8H_5F_{13}O_3S$	426.9668	M6:2 FtS
8:2 Fluorotelomer sulfonic acid	8:2 FTS	$C_{10}H_5F_{17}O_3S$	526.9604	M6:2 FtS
<u>Fluorotelomer phosphate diesters</u>				
6:2 Fluorotelomer phosphate diester	6:2 diPAP	$C_{16}H_9F_{26}O_4P$	788.9739	M4-6:2-diPAP
8:2 Fluorotelomer phosphate diester	8:2 diPAP	$C_{20}H_9F_{34}O_4P$	988.9597	M4-8:2-diPAP
6:2/8:2 Fluorotelomer phosphate diester	6:2/8:2 diPAP	$C_{18}H_9F_{30}O_4P$	888.9675	M4-8:2-diPAP
<u>Perfluorooctane sulfonamide-based substances</u>				
Perfluorooctane sulfonamide	FOSA	$C_8H_2F_{17}NO_2S$	497.9451	d3-MeFOSA
N-Methyl perfluorooctane sulfonamide	MeFOSA	$C_9H_4F_{17}NO_2S$	511.9608	d3-MeFOSA
N-Ethyl perfluorooctane sulfonamide	EtFOSA	$C_{10}H_6F_{17}NO_2S$	525.9764	d3-MeFOSA
Perfluorooctane sulfonamidoacetic acid	FOSAA	$C_{10}H_4F_{17}NO_4S$	555.9506	d3-MeFOSAA
N-Methyl perfluorooctane sulfonamidoacetic acid	MeFOSAA	$C_{11}H_6F_{17}NO_4S$	569.9662	d3-MeFOSAA
N-Ethyl perfluorooctane sulfonamidoacetic acid	EtFOSAA	$C_{12}H_8F_{17}NO_4S$	583.9819	d3-MeFOSAA
<u>Replacements and "others"</u>				
Heptafluoropropoxy propanoic acid	HFPO-DA (GenX)	$C_6HF_{11}O_3$	284.9761	M3-HFPO-DA
Perfluoroethylcyclohexane sulfonic acid	PFECHS	$C_8HF_{15}O_3S$	460.9323	MPFOS
9Cl-Perfluoro-3-oxononane sulfonic acid	9Cl-PF3ONS	$C_8HClF_{16}O_4S$	530.8945	MPFOS
11Cl-Perfluoro-3-oxoundecane sulfonic acid	11Cl-PF3OUdS	$C_{10}HClF_{20}O_4S$	630.8881	MPFOS
7H-Perfluoroheptanoic acid	7H-PFHpA	$C_7H_2F_{12}O_2$	344.9779	MPFHpA
Ammonium perfluoro-4.8-dioxa-3H-nonanoic acid	ADONA	$C_7H_5F_{12}NO_4$	376.9678	MPFOA

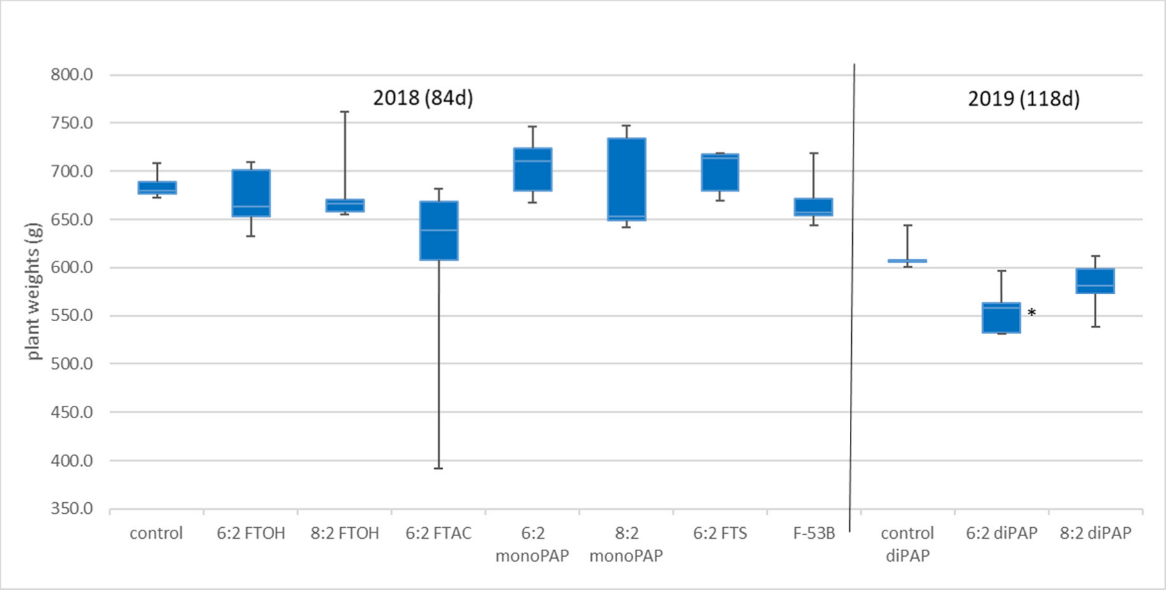

*Figure S1: Weights of maize plants (in g) in two pot experiments (2018 with 84 days and 2019 with 118-day growth period) with spiked PFAS-precursors and F-53B. Results shown as boxplots with the first and third quartile in blue boxes. Whiskers express minimum and maximum values, respectively. *significant difference to the control.*

Table S3: Limits of Quantification (LOQ) for precursors in soil and plant material of the pot study with maize. LOQ for PFAAs were 0.5 µg/kg in soil and plant material. n.a. = not applicable.

Treatment (µg/kg)	6:2 FTOH	8:2 FTOH	6:2 FTAC	6:2 mono PAP	8:2 mono PAP	6:2 diPAP	8:2 diPAP	F-53B
6:2 FTOH plant	< 5	< 20	< 15	n.a.	n.a.	< 0.5	< 0.5	< 0.5
6:2 FTOH soil	< 5	< 5	< 10	n.a.	n.a.	< 0.5	< 0.5	< 0.5
8:2 FTOH plant	< 3	< 25	< 20	n.a.	n.a.	< 0.5	< 0.5	< 0.5
8:2 FTOH soil	< 5	< 5	< 10	n.a.	n.a.	< 0.5	< 0.5	< 0.5
6:2 monoPAP	< 0.5	< 0.5	< 0.5	n.a.	n.a.	< 0.5	< 0.5	< 0.5
8:2 monoPAP	< 0.5	< 0.5	< 0.5	n.a.	n.a.	< 0.5	< 0.5	< 0.5
6:2 diPAP plant	< 0.5	< 0.5	< 0.5	n.a.	n.a.	< 0.5	< 0.5	< 0.5
6:2 diPAP soil	< 0.5	< 0.5	< 0.5	n.a.	n.a.	< 0.5	< 0.5	< 0.5
8:2 diPAP plant	< 0.5	< 0.5	< 0.5	n.a.	n.a.	< 0.5	< 0.5	< 0.5
8:2 diPAP soil	< 0.5	< 0.5	< 0.5	n.a.	n.a.	< 0.5	< 0.5	< 0.5
6:2 FTAC plant	< 10	< 30	21	n.a.	n.a.	< 0.5	< 0.5	< 0.5
6:2 FTAC soil	< 5	< 5	< 10	n.a.	n.a.	< 0.5	< 0.5	< 0.5
F-53B plant	< 0.5	< 0.5	< 0.5	n.a.	n.a.	< 0.5	< 0.5	< 0.5
F-53B soil	< 0.5	< 0.5	< 0.5	n.a.	n.a.	< 0.5	< 0.5	< 0.5

Table S4: Concentration rate (CR), transfer rate (TR), conversion rate (CVR), and recovery rate (RR) of PFAS-precursors and degradation products in the pot study with maize

precursor treatment	CR/TR (%)	PFBA	PFPeA	PFHxA	PFHpA	PFOA	PFNA	PFBS	CVR	6:2 Cl-PFESA	8:2 Cl-PFESA	6:2 diPAP	8:2 diPAP	RR_{Prec}	RR_{total}
6:2 FTOH	CR_{soil}	0.32	2.53	2.99	0.00	0.00	0.00	0.00	5.83	0.00	0.00	0.00	0.00	0.00	5.83
	TR_{stem}	0.01	0.01	0.01	0.00	0.00	0.00	0.00	0.03	0.00	0.00	0.00	0.00	0.00	0.03
	TR_{leaf}	0.06	0.08	0.01	0.00	0.00	0.00	0.00	0.15	0.00	0.00	0.00	0.00	0.00	0.15
	TR_{cob}	0.00	0.00	0.00	0.00	0.00	0.00	0.00	0.01	0.00	0.00	0.00	0.00	0.00	0.01
	TR_{shoot}	0.07	0.09	0.02	0.00	0.00	0.00	0.00	0.18	0.00	0.00	0.00	0.00	0.00	0.18
8:2 FTOH	CR_{soil}	0.17	0.51	0.96	2.62	6.65	0.00	0.00	10.90	0.00	0.00	0.00	0.00	0.00	10.90
	TR_{stem}	0.01	0.00	0.00	0.00	0.00	0.00	0.00	0.02	0.00	0.00	0.00	0.00	0.00	0.02
	TR_{leaf}	0.03	0.02	0.01	0.00	0.00	0.00	0.00	0.07	0.00	0.00	0.00	0.00	0.00	0.07
	TR_{cob}	0.00	0.00	0.00	0.00	0.00	0.00	0.00	0.00	0.00	0.00	0.00	0.00	0.00	0.00
	TR_{shoot}	0.04	0.02	0.01	0.01	0.01	0.00	0.00	0.08	0.00	0.00	0.00	0.00	0.00	0.08
6:2 monoPAP	CR_{soil}	0.31	2.60	3.22	0.00	0.00	0.00	0.00	6.14	0.00	0.00	0.00	0.00	0.00	6.14
	TR_{stem}	0.02	0.03	0.01	0.00	0.00	0.00	0.00	0.06	0.00	0.00	0.00	0.00	0.00	0.06
	TR_{leaf}	0.11	0.27	0.03	0.00	0.00	0.00	0.00	0.41	0.00	0.00	0.00	0.00	0.00	0.41
	TR_{cob}	0.00	0.01	0.00	0.00	0.00	0.00	0.00	0.01	0.00	0.00	0.00	0.00	0.00	0.01
	TR_{shoot}	0.13	0.31	0.04	0.00	0.00	0.00	0.00	0.48	0.00	0.00	0.00	0.00	0.00	0.48
8:2 monoPAP	CR_{soil}	0.00	0.00	0.39	0.93	5.23	0.00	0.00	6.56	0.00	0.00	0.00	0.00	0.00	6.56
	TR_{stem}	0.01	0.00	0.00	0.00	0.00	0.00	0.00	0.01	0.00	0.00	0.00	0.00	0.00	0.01
	TR_{leaf}	0.03	0.02	0.00	0.00	0.00	0.00	0.00	0.05	0.00	0.00	0.00	0.00	0.00	0.05
	TR_{cob}	0.00	0.00	0.00	0.00	0.00	0.00	0.00	0.00	0.00	0.00	0.00	0.00	0.00	0.00
	TR_{shoot}	0.03	0.02	0.01	0.00	0.00	0.00	0.00	0.06	0.00	0.00	0.00	0.00	0.00	0.06
6:2 diPAP	CR_{soil}	0.41	1.72	1.75	0.01	0.00	0.00	0.00	3.90	0.00	0.00	13.63	0.00	13.63	17.53
	TR_{root}	0.03	0.32	0.13	0.00	0.00	0.00	0.00	0.48	0.00	0.00	0.03	0.00	0.03	0.52
	TR_{stem}	0.04	0.32	0.06	0.00	0.00	0.00	0.00	0.42	0.00	0.00	0.00	0.00	0.00	0.42
	TR_{leaf}	0.24	0.28	0.23	0.00	0.00	0.00	0.00	0.75	0.00	0.00	0.00	0.00	0.00	0.75
	TR_{ears}	0.01	0.12	0.02	0.00	0.00	0.00	0.00	0.15	0.00	0.00	0.00	0.00	0.00	0.15
	TR_{cob}	0.01	0.02	0.00	0.00	0.00	0.00	0.00	0.04	0.00	0.00	0.00	0.00	0.00	0.04
	$TR_{kernels}$	0.00	0.05	0.02	0.00	0.00	0.00	0.00	0.07	0.00	0.00	0.00	0.00	0.00	0.07
	TR_{cob}	0.03	0.19	0.05	0.00	0.00	0.00	0.00	0.26	0.00	0.00	0.00	0.00	0.00	0.26
	TR_{shoot}	0.31	0.79	0.33	0.00	0.00	0.00	0.00	1.44	0.00	0.00	0.00	0.00	0.00	1.44
	TR_{plant}	0.36	1.31	0.51	0.00	0.00	0.00	0.00	2.18	0.00	0.00	0.04	0.00	0.04	2.22

Table S4 continued

treatment	CR/TR (%)	PFBA	PFPeA	PFHxA	PFHpA	PFOA	PFNA	PFBS	CVR	6:2 Cl-PFESA	8:2 Cl-PFESA	6:2 diPAP	8:2 diPAP	RR_{Prec}	RR_{total}
8:2 diPAP	CR_{soil}	0.13	0.17	0.42	2.34	20.23	0.00	0.00	23.29	0.00	0.00	0.00	43.20	43.20	66.50
	TR_{root}	0.00	0.01	0.01	0.02	0.14	0.00	0.00	0.18	0.00	0.00	0.00	0.13	0.13	0.31
	TR_{stem}	0.05	0.05	0.01	0.01	0.02	0.00	0.00	0.13	0.00	0.00	0.00	0.00	0.00	0.13
	TR_{leaf}	0.13	0.13	0.03	0.03	0.07	0.00	0.00	0.38	0.00	0.00	0.00	0.01	0.01	0.39
	TR_{ears}	0.00	0.01	0.00	0.00	0.00	0.00	0.00	0.01	0.00	0.00	0.00	0.00	0.00	0.01
	TR_{cob}	0.00	0.00	0.00	0.00	0.00	0.00	0.00	0.00	0.00	0.00	0.00	0.00	0.00	0.00
	$TR_{kernels}$	0.00	0.00	0.00	0.00	0.00	0.00	0.00	0.00	0.00	0.00	0.00	0.00	0.00	0.00
	TR_{cob}	0.00	0.01	0.00	0.00	0.00	0.00	0.00	0.02	0.00	0.00	0.00	0.00	0.00	0.02
	TR_{shoot}	0.18	0.19	0.04	0.04	0.09	0.00	0.00	0.53	0.00	0.00	0.00	0.01	0.01	0.54
	TR_{plant}	0.19	0.20	0.06	0.06	0.23	0.00	0.00	0.73	0.00	0.00	0.00	0.14	0.14	0.87
6:2 FTAC	CR_{soil}	0.17	0.41	0.63	0.00	0.00	0.00	0.00	1.20	0.00	0.00	0.00	0.00	0.00	1.20
	TR_{stem}	0.00	0.00	0.00	0.00	0.00	0.00	0.00	0.01	0.00	0.00	0.00	0.00	0.00	0.01
	TR_{leaf}	0.02	0.04	0.01	0.00	0.00	0.00	0.00	0.07	0.00	0.00	0.00	0.00	0.00	0.07
	TR_{cob}	0.00	0.00	0.00	0.00	0.00	0.00	0.00	0.00	0.00	0.00	0.00	0.00	0.00	0.00
	TR_{shoot}	0.03	0.04	0.01	0.00	0.00	0.00	0.00	0.08	0.00	0.00	0.00	0.00	0.00	0.08
F-53B	CR_{soil}	0.00	0.00	0.00	0.00	0.00	0.00	0.00	0.00	82.15	52.62	0.00	0.00	134.78	134.78
	TR_{stem}	0.00	0.00	0.00	0.00	0.00	0.00	0.00	0.00	0.02	0.00	0.00	0.00	0.02	0.02
	TR_{leaf}	0.00	0.00	0.00	0.00	0.00	0.00	0.00	0.00	0.04	0.00	0.00	0.00	0.04	0.04
	TR_{cob}	0.00	0.00	0.00	0.00	0.00	0.00	0.00	0.00	0.00	0.00	0.00	0.00	0.00	0.00
	TR_{shoot}	0.00	0.00	0.00	0.00	0.00	0.00	0.00	0.00	0.07	0.00	0.00	0.00	0.07	0.07
